# The Principle of Introducing Halogen Ions Into β-FeOOH: Controlling Electronic Structure and Electrochemical Performance

**DOI:** 10.1007/s40820-020-00440-2

**Published:** 2020-05-06

**Authors:** Dongbin Zhang, Xuzhao Han, Xianggui Kong, Fazhi Zhang, Xiaodong Lei

**Affiliations:** State Key Laboratory of Chemical Resource Engineering, Beijing University of Chemical Technology, PO Box 98, Beijing, 100029 People’s Republic of China

**Keywords:** β-FeOOH, Halogen ion embedment, Tuning electronic structure, Supercapacitor performance

## Abstract

Coordination tuning electronic structure of host materials is a quite effective strategy for activating and improving the intrinsic properties. Herein, halogen anion (X^−^)-incorporated β-FeOOH (β-FeOOH(X), X = F^−^, Cl^−^, and Br^−^) was investigated with a spontaneous adsorption process, which realized a great improvement of supercapacitor performances by adjusting the coordination geometry. Experiments coupled with theoretical calculations demonstrated that the change of Fe–O bond length and structural distortion of β-FeOOH, which is rooted in halogen ions embedment, led to the relatively narrow band gap. Because of the strong electronegativity of X^−^, the Fe element in β-FeOOH(X)s presented the unexpected high valence state (3 + *δ*), which is facilitating to adsorb SO_3_^2−^ species. Consequently, the designed β-FeOOH(X)s exhibited the good electric conductivity and enhanced the contact between electrode and electrolyte. When used as a negative electrode, the β-FeOOH(F) showed the excellent specific capacity of 391.9 F g^−1^ at 1 A g^−1^ current density, almost tenfold improvement compared with initial β-FeOOH, with the superior rate capacity and cyclic stability. This combinational design principle of electronic structure and electrochemical performances provides a promising way to develop advanced electrode materials for supercapacitor.

**Electronic supplementary material:**

The online version of this article (10.1007/s40820-020-00440-2) contains supplementary material, which is available to authorized users.

## Highlights

- Halogen ion-incorporated β-FeOOH(X)s (X = F^−^, Cl^−^, Br^−^) were designed and fabricated successfully and showed good performance for negative electrodes of supercapacitors.
- The embedment of X^−^ caused the change of Fe–O bond length and structural distortion of β-FeOOH, which resulted in the narrow band gap and good electric conductivity.
- The presence of unexpected high valence state (3 + *δ*) Fe element facilitated the adsorption for SO_3_^2−^ species endowing the β-FeOOH(X)s with good wettability in Na_2_SO_3_ electrolyte.

**Electronic supplementary material:**

The online version of this article (10.1007/s40820-020-00440-2) contains supplementary material, which is available to authorized users.

## Graphical abstract

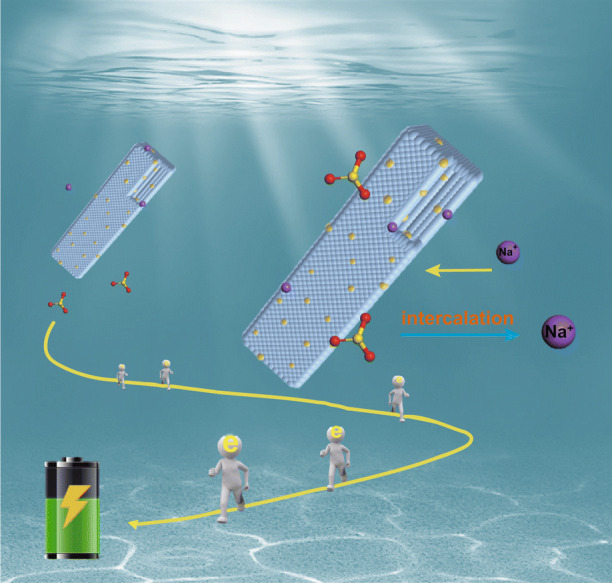

## Introduction

Supercapacitors are a type of energy storage and conversion device which have attracted considerable attention from both academia and industry by virtue of their high power density, long lifespan, rapid charging/discharging rate, and low maintenance cost [1, 2]. However, the energy density of supercapacitors is much lower than that of Li-ion batteries, which limits its application on fulfilling the requirements of large-scale energy applications [3, 4]. According to the energy density equation (*E *= 1/2CV^2^), it is meaningful to increase the specific capacity and potential windows of both positive and negative electrodes. In the past few years, though the positive electrode materials have a rapid development, the sluggish research on negative electrode materials has hindered the energy density improvement [1–6]. Iron-based materials including Fe_3_O_4_, Fe_2_O_3_ and FeOOH, etc. [3, 7–11], as the promising negative electrode materials, have the high specific capacity in theory due to its charge storage mechanism based on redox reactions [12–16]. However, the poor electric conductivity and low intrinsic activity of iron-based materials limit the electron transfer efficiency and lead to the disappointing supercapacitor performance in practice [7, 12, 13, 17]. Therefore, it is urgent and significant to break through the barrier between superior theoretical capacity and inferior actual performance of iron-based materials [18].

Defect engineering such as cation and vacancy doping is considered as a feasible method that can conquer this challenge, because of its effect on controlling the electronic structure of host materials [19–26]. For example, it was found that the introduction of Co cations into FeOOH can give rise to unusual enhancement of oxygen evolution reaction activity [27]. After the introduction of O vacancies (*V*_o_) into Fe_2_O_3_, the obtained material showed the good catalyst capacity for SO_3_^2−^ species to realize the high-energy-storage property [28]. However, how to realize the modification of material electronic structure by incorporation of anions is rarely reported and an urgent task needs to be addressed and resolved [29]. As a matter of fact, because of the convenient operation and unique modulate manner of anions for host materials, forming coordination bonding, the host materials can produce abundant vacancies and keep the integrity of crystal structure well; thus, they exhibit excellent performances [30–33].

Among all anion categories, halogen anions have the strongest electronegativity and abundant p-orbit electrons [34]. Their coordination doping easy causes variation of electronic structure of host material to improve the electrochemical performance [29, 34, 35]. In this work, we focused on the principle of anions embedment into β-FeOOH and fabricated some halogen anions, including F^−^, Cl^−^, and Br^−^, doping β-FeOOH materials. By conducting the elaborate structure analyses and supercapacitor performance tests of β-FeOOH and β-FeOOH(X)s, we built a bridge between the electronic structure regulation and electrochemical performance. The results showed that the introduction of X^−^ ions led to the Fe–O bond length changed and structural distortion of β-FeOOH. The band gap narrowed after X^−^ ions doping indicated the promotion of electric conductivity. Because of the strong electronegativity of X^−^, the Fe element in β-FeOOH(X)s presented the unexpected high valence state (3 + *δ*), which is facilitating to the contact between electrode and electrolyte, showing good wettability. Benefiting from these unique characteristics, the β-FeOOH(X)s exhibited the superior supercapacitor performances.

## Experimental Sections

### Materials and Reagents

The Nafion solution (5 wt%) was obtained from Du Pont China Holding Co., Ltd. The nickle foam and copper foam were purchased from Suzhou Jia Shi De metal foam Co., Ltd. (Suzhou China). The other reagents (analytical grade) in the experiments were obtained from Sinopharm Chemical Reagents Co., Ltd. China, and used without any further purification.

### Synthesis of β-FeOOH

The β-FeOOH was obtained by a hydrothermal synthesis method. 0.404 g Fe(NO_3_)_3_·9H_2_O with 0.3 g urea was dissolving into 30 mL deionized water with magnetic stirring. Then, the mixture was transferred to a 50-mL Teflon-lined stainless-steel autoclave and maintained at 120 °C for 6 h. The product was removed and washed three times with deionized water and dried at 60 °C.

### Synthesis of β-FeOOH(X)

To fabricate the designed β-FeOOH(X) samples. 50 mg β-FeOOH was dispersed in 10 mL 1 mol L^−1^ NaX aqueous solution (including NaF, NaCl, NaBr, and NaI) with magnetic stirring for 24 h at room temperature. Then, the product was removed and washed three times with deionized water and dried at 60 °C to obtain β-FeOOH(X) samples. The Fe-normalized chemical formulas of the obtained β-FeOOH(X)s are FeOOH,FeO_0.613_(OH)_1.387_F_0.387_, FeO_0.641_(OH)_1.359_Cl_0.359_, and FeO_0.654_(OH)_1.346_Br_0.346_, respectively.

### Synthesis of NiCo Hydroxides/Cu(OH)_2_/CF

The NiCo hydroxides/Cu(OH)_2_/CF (NCF) was obtained according our previous work with some modifications [36]. Briefly, copper foam was used as substrate to form Cu(OH)_2_ nanowires array with alkaline oxidative etchant solution (AOES). Then, the Cu(OH)_2_/CF was used as working electrode to fabricate hollow tubular NiCo hydroxides/Cu(OH)_2_ nanoarray by electrochemical synthesis method. To satisfy the demand of charge matching (*Q*_+_ = *Q*_−_), the time of electrodeposition was controlled, and the mass loading of active materials was ~ 1 mg cm^−2^.

### Materials Characterization

X-ray diffraction (XRD) patterns were collected on a Rigaku XRD-6000 diffractometer using Cu Kα radiation, from 10° to 80°, with the scan rate of 10° min^−1^. The Raman spectra were recorded over the wavelength range from 100 to 1000 cm^−1^ with 514-nm laser excitation. The solid UV–Vis absorption spectra were recorded over the range from 200 to 700 nm. The contact angle tests were investigated in 1 mol L^−1^ Na_2_SO_3_ aqueous solution. The morphology of samples was investigated using a scanning electron microscope (SEM; Zeiss SUPRA 55) with an accelerating voltage of 20 kV, combined with energy-dispersive X-ray spectroscopy (EDS). High-resolution transmission electron microscopy (HRTEM) images were recorded using a JEOL JEM-2010 field-emission transmission electron microscope with an accelerating voltage of 200 kV. X-ray photoelectron spectroscopy (XPS) measurements were taken on a Thermo VG ESCALAB 250 X-ray photoelectron spectrometer with Al Kα radiation at a pressure of about 2 × 10^−9^ Pa.

### Electrochemical Performance Tests

To carry out the electrochemical performance tests, the slurry was prepared by mixing 2 mg active material (as-prepared β-FeOOH and β-FeOOH(X)s), 45 μL of Nafion solution (5 wt%, DuPont), and 1 mL anhydrous ethanol. The mixture was ultrasonicated for 5 min to get a homogeneous suspension. Then, the slurry was dripped onto the prepared Ni foam to obtain the working electrode, leading to an active loading of ~ 2 mg cm^−2^. Finally, the as-prepared working electrodes were dried at 60 °C for 1 h.

The electrochemical measurements were taken on an electrochemical workstation (CHI 660E, CH Instruments Inc, Chenhua, Shanghai) with the three-electrode system in 1 mol L^−1^ Na_2_SO_3_ aqueous solution as the electrolyte. A platinum wire was used as counter electrode, saturated calomel electrode (SCE) electrode was the reference electrode, and the obtained samples were the working electrodes. The electrochemical performance measurements were taken as follows: The cyclic voltammetry (CV) experiments were performed at different scanning rates (from 5 to 100 mV s^−1^). The galvanostatic charge/discharge (GCD) measurements were taken within the potential window from 0 to − 1.1 V at various current densities (from 1 to 10 A g^−1^). The cyclic stabilities were tested by repeat 2000 charge/discharge cycles at the current density of 10 A g^−1^. The electrochemical impedance spectroscopy (EIS) was carried out by applying an AC voltage with 5 mV amplitude in the frequency range from 100 kHz to 0.01 Hz. During the testing, the samples were used as negative electrodes.

The dual electrolyte asymmetric supercapacitor (DESC) was assembled when the β-FeOOH(F) was used as the negative electrode and NCF as the positive electrode, and tested in H-type of electrolytic cell, while 1 mol L^−1^ Na_2_SO_3_ aqueous solution and 1 mol L^−1^ KOH aqueous solution were used as the negative and positive electrode electrolytes, respectively. The electrochemical performance measurements were taken as follows: The cyclic voltammetry (CV) experiments were performed at different scanning rates (from 5 to 100 mV s^−1^). The galvanostatic charge/discharge (GCD) measurements were taken within the potential window from 0 to 1.5 V at various current densities (from 1 to 10 A g^−1^). The cyclic stabilities were tested by repeat 2000 charge/discharge cycles at the current density of 10 A g^−1^. The electrochemical impedance spectroscopy (EIS) was carried out by applying an AC voltage with 5 mV amplitude in the frequency range from 100 kHz to 0.01 Hz.

### Theoretical Calculation

Plane-wave density functional theory (DFT) + U calculations of the electronic properties of β-FeOOH systems were carried out using the CASTEP module in Materials Studio. GGA with a PBE functional was employed for the DFT exchange correlation energy. The Brillouin zone was sampled by 1 × 1 × 1 k-points. The values of *U* − *J* (Ueff) were 5.00 eV for Fe. The core electrons were performed with the ultrasoft pseudopotentials to improve transferability. The energy cut-off of 380 eV was applied for the plane wave truncation. The value of self-consistent field (SCF) tolerance was 1 × 10^−4^ eV/atom. In addition, the plains of crystal surfaces were selected and calculated based on the results of HRTEM.

The adsorption energy, *E*_ads_, is defined as:

1 $$E_{\text{ads}} = E\left( {{\text{facet}}/{\text{SO}}_{3}^{2 - } } \right) \, - E\left( {\text{facet}} \right) \, - E\left( {{\text{SO}}_{3}^{2 - } } \right)$$

where *E*(facet/SO_3_^2−^) is the total energy of the optimized adsorption structure, *E*(SO_3_^2−^) is the energy of SO_3_^2−^, and *E*(facet) is the energy of optimized β-FeOOH (310) facet, respectively. Meanwhile, the adsorption energy for X^−^ (F^−^, Cl^−^, Br^−^) is also calculated according to Eq. 1, while the SO_3_^2−^ is replaced by X^−^. Generally, the *E*_ads_ is more negative, indicating that the adsorption process is more likely to occur spontaneously attributed to the attractive interaction.

## Results and Discussion

### Morphology and Structure Characterization of β-FeOOH(X)s

**Fig. 1:**
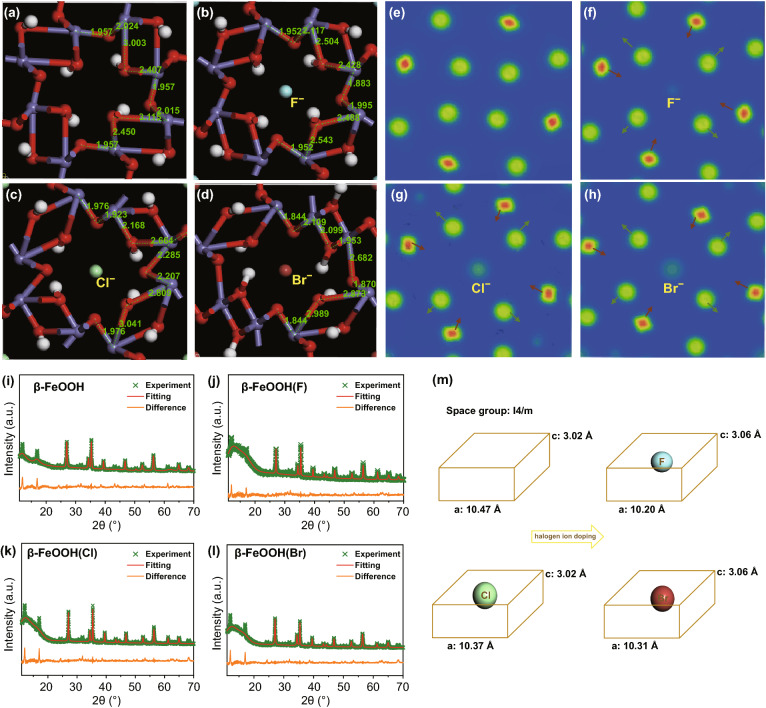
**a–d** DFT calculation, **e–h** the corresponding charge density differential diagram, and **i–l** Rietveld XRD patterns of β-FeOOH and β-FeOOH(X)s. **m** Corresponding schematic illustration of the contraction of β-FeOOH cell after doping halogen ions

The crystal structure of β-FeOOH is shown in Fig. S1a. Obviously, the obtained β-FeOOH has typical open-framework structure with the diameter of aperture ~ 4.194 Å, larger than the radius of halogen anions (for F^−^ is 1.16, Cl^−^ is 1.64, and Br^−^ is 1.80 Å. See Fig. S1b and Table S1). The binding energies of incorporating halogen anions into β-FeOOH were simulated by DFT calculations. The β-FeOOH(X)s showed the lower energy than that of β-FeOOH, indicating the more stable structure of β-FeOOH(X)s than initial β-FeOOH. The results confirmed the behavior of introducing halogen anions into β-FeOOH is a spontaneous process (Fig. S1c). However, because of the strong reducing property of I^−^ ions, it is difficult to construct β-FeOOH(I) material (Table S2 and Fig. S2). In our expectation, the introduction of halogen anion into β-FeOOH led to the microstructure change, including bond length, bond angle, and electric charge density. According to the geometry optimization datum, the bond lengths of Fe–Os (marked from Fe–O1 to Fe–O8) have different variation tendency, showing the structural distortion of β-FeOOH after the embedment of X^−^ anions (Fig. 1a–d and Table S3). This evolution is illustrated by charge density differential diagram as shown in Fig. 1e–h. Because of the electronegativity of halogen anions and electrostatic interaction between X^−^ and β-FeOOH, the distance between Fe^3+^ and X^−^ reduces and that between O^2−^ and X^−^ increases. Therefore, the interaction between X^−^ and the FeO_6_ units will increase and allow the lattice to contract slightly to increase the degree of structural distortion. The mechanism of structure evolution was further analyzed using crystal field theory as discussion in the later section. As usual, the structural distortion causes the change of electronic structure of material, which affects the electric conductibility and wettability of the material in electrolyte, further influencing its electrochemical performances [25, 37–39]. These premise conditions provide the possibility to investigating the regular effects of halogen anions for β-FeOOH on electronic structure and electrochemical performance.

Based on the discussion above, the β-FeOOH was prepared by one-step hydrothermal method. The detail fabrication information was given in SI. Then, the as-prepared β-FeOOH was dispersed into corresponding NaX aqueous solution to obtain the β-FeOOH(X) samples. The presence of diffraction peaks of (110), (310), (211), and (521) at 12.0°, 27.0°, 35.5°, and 56.3°, respectively, showed the successful formation of β-FeOOH (JCPDS No. 34-1266). After the incorporation of halogen anions, though the characteristic diffraction peaks of β-FeOOH remained, the relatively small shifts of (110), (310), and (211) planes reflected the bond length of Fe–O changed and the lattice distortion of β-FeOOH (Fig. S3). The Rietveld refinement along with the experimental XRD patterns of β-FeOOH(X)s is shown in Fig. 1i–l. The Rietveld refinement with a space group of I4/m reveals that X^−^ occupied the center of 2 × 2 tunnels of β-FeOOH [40]. After the introduction of X^−^, the unit cell size has a slight contraction (Fig. 1m), which is consistent with our speculation with DFT calculations.

As seen in Fig. S4a–d, the β-FeOOH shows the typical nanorod structure and has no obvious variation before and after the embedment of halogen anion process. The mapping EDS tests were applied to detect the existence of F^−^, Cl^−^, and Br^−^ (Fig. S4e–h). The amount of halogen anions in corresponding β-FeOOH(X) was 13.7% for F^−^, 10.67% for Cl^−^, and 10.63% for Br^−^, respectively (Table S4). All of them dispersed on the surface of β-FeOOH nanorod uniformly. These results further confirmed the successful fabrication of halogen anion-embedded β-FeOOH. Additionally, the HRTEM images are shown in Fig. S4i–p. The clear lattice fringe with a spacing of ~ 0.33 nm was observed, corresponding to the (310) plane of β-FeOOH. Interestingly, after the embedment of halogen anions, the lattice distance of (310) plane decreased from 0.330 to 0.329 nm, which was consistent with the XRD results (Fig. S3c). It is worth mentioning that no matter what kinds of halogen anions embedded, the fabricated materials exhibited the single crystal structure (inset in Fig. S4m–p).

### Electrochemical Performances of β-FeOOH(X)s

**Fig. 2:**
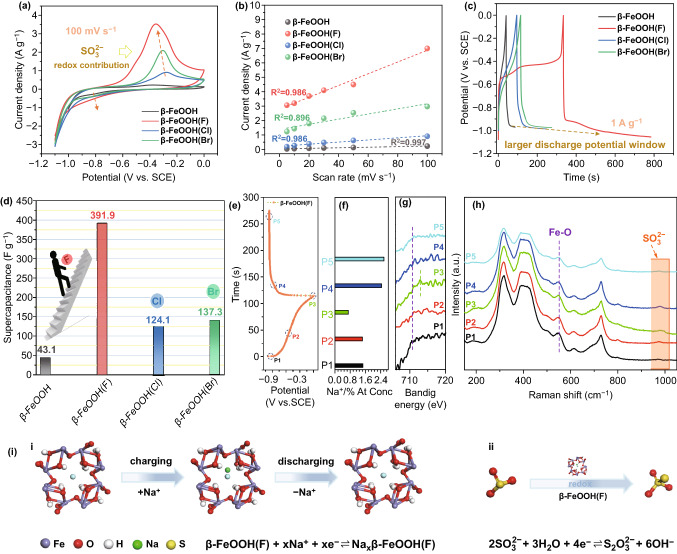
Electrochemical performances of β-FeOOH and β-FeOOH(X)s. **a** CVs, **b** plots of peak current density versus scan rate, **c** GCDs, **d** comparison of specific capacity, **e–h** ex situ XPS and Raman of β-FeOOH(F) under different charge/discharge potentials, and **i** scheme of energy storage mechanism of β-FeOOH(F)

Electrochemical performances of the fabricated materials including β-FeOOH and β-FeOOH(X)s were performed in 1 mol L^−1^ Na_2_SO_3_ electrolyte. Two characteristic redox peaks were detected in CV curves at ~ − 0.38 and ~ − 0.80 V, which was contributed to the redox reaction of SO_3_^2−^/S_2_O_3_^2−^ during the charge/discharge process (Figs. 2a and S5). Obviously, the peak current density of β-FeOOH(F) was larger than that of β-FeOOH(Br), β-FeOOH(Cl), and β-FeOOH, confirming the β-FeOOH(F) had the strongest catalytic capacity for redox reaction of SO_3_^2−^ species. Meanwhile, the peak current density plots versus scan rate from 5 to 100 mV s^−1^ are shown in Fig. 2b. β-FeOOH and β-FeOOH(X)s showed linear relationship between peaks current density and scan rate, indicating the energy storage behavior on the surface of electrodes was an electrochemical-controlled process instead of diffusion-controlled process [41, 42]. This is the premise of high rate capacity as displayed in Fig. S6a. In detail, when the current density increased from 1 to 10 A g^−1^, the specific capacitance of β-FeOOH(F) decreased from 391.9 to 275.0 F g^−1^, resulting in the rate capacity was 70.17%, better than that of β-FeOOH(Br) (60.45%), β-FeOOH(Cl) (61.24%), and β-FeOOH (26.63%). The specific capacitances of samples were calculated by GCD tests, the results showed that the specific capacitance of β-FeOOH(F) had ~ tenfold improvement compared with that of initial β-FeOOH, and even the β-FeOOH(Cl) and β-FeOOH(Br) also showed the great enhancement (Fig. 2c, d). The specific capacitances of β-FeOOH(F) are comparable to the values reported in the previous literature. (Table S5) After the 2000 charge/discharge cycles, the β-FeOOH(F) showed the better cyclic stability than that of others (Fig. S6b). Meanwhile, there was no signal of dissolved Fe element detected in electrolyte (Table S6), confirming the good stability of samples, which is also verified by XRD and XPS results (Fig. S7).

To investigate the electrochemical energy storage mechanism, the β-FeOOH(F) electrode under different charge/discharge potentials was studied by ex situ XRD, XPS, and Raman tests (Fig. 2e–h). As is shown for P3 to P5, on account of the Na^+^ insertion, lattice structure expanded, and the peak of (310) plane shifted toward low degree. The insertion of Na^+^ ion resulted in reduction in the valence state of Fe element and showed the red shift reflection in XPS analysis. In contrast, for P1 to P3, the peak of (310) plane shifted toward high degree and reflected blue shift in XPS, indicating the lattice contraction and valence state of Fe element increased after Na^+^ extraction (Fig. S8 and Table S7). The intensity and width of Fe–O peak at ~ 550 cm^−1^ in Raman spectra changed during the charge/discharge process, confirming the length of Fe–O bond changed, due to the structure expansion/contraction caused by the Na^+^ ions insertion/extraction (Fig. 2h). These results confirmed the insertion/extraction behavior of Na^+^ ions during charge/discharge process in Na_2_SO_3_ electrolyte [43–45]. In addition, an obvious peak at ~ 980 cm^−1^ assigned to SO_3_^2−^ was detected at P3 potential but hardly founded at P1 and P5, indicating the SO_3_^2−^ adsorbed on the surface of electrode and took place redox reaction from SO_3_^2−^ to S_2_O_3_^2−^ [28, 44]. Summing up, the electrochemical energy storage mechanism of β-FeOOH(F) electrode can be divided into two ways (Fig. 2i). The first one is the Na^+^ ions insertion/extraction reaction. And the second one is the SO_3_^2−^ electrolyte redox reaction catalyzed by β-FeOOH(F). Because both the electrochemical energy storage reactions occurred together, the designed β-FeOOH(F) showed the excellent supercapacitor negative electrode performances.

### Effect of Halogen Anions Embeding

**Fig. 3:**
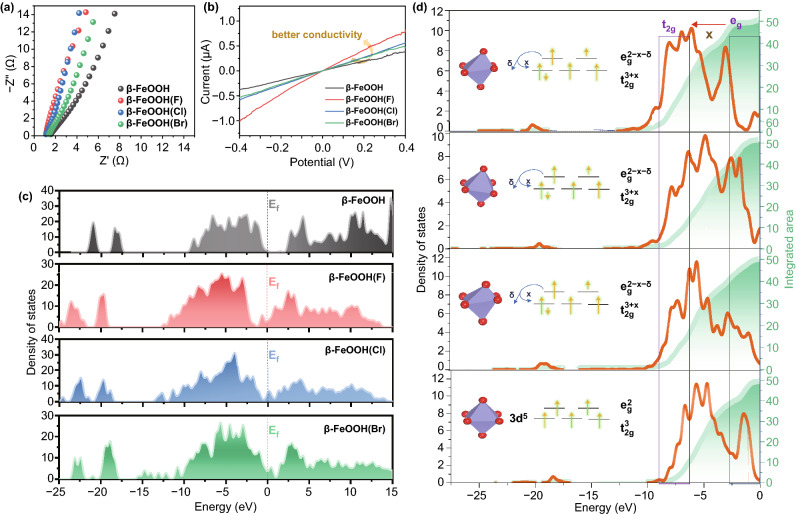
**a** EIS, **b** *I*–*V*, **c** DOS curves, and **d** PDOS of Fe 3*d* valance band orbitals of β-FeOOH and β-FeOOH(X)s

**Fig. 4:**
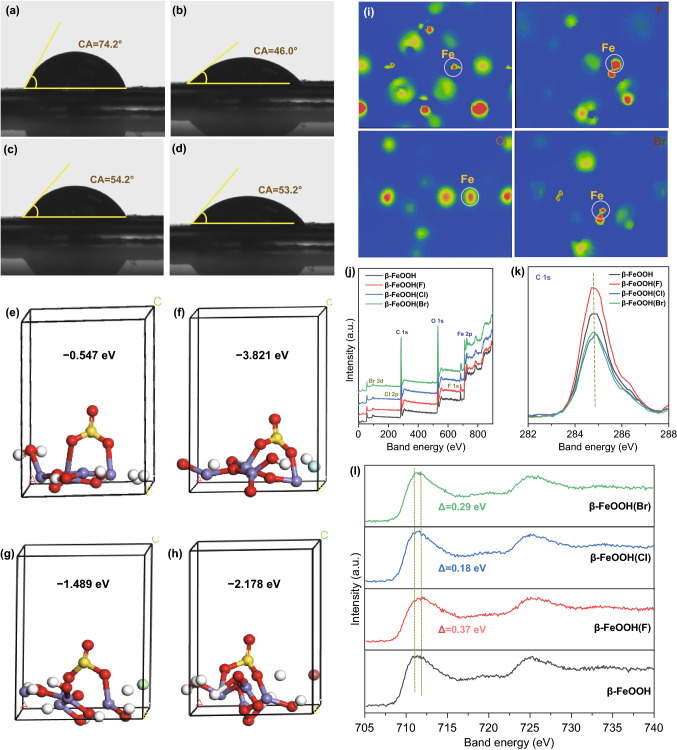
**a**–**d** Contact angles, **e–h** adsorption energies for SO_3_^2−^, **i** charge density differential diagram, **j–l** XPS of β-FeOOH and β-FeOOH(X)s

The factor for boosting of electrochemical performances of β-FeOOH after X^−^ ions embedment was further investigated as shown in Fig. 3. The good electric conductivity is necessary for guaranteeing the rapid electron transfer rate when designing an electrode material, which improves electron utilization efficiency [46, 47]. According to the EIS and *I*–*V* curves, the β-FeOOH(X)s exhibited the better electric conductivity than that of initial β-FeOOH. Among them, the β-FeOOH(F) showed the best electric conductivity (Fig. 3a, b). These results were consistent with the DFT calculations. From Fig. 3c, we found that the total density of states (TDOS) curve of β-FeOOH showed obvious band gap at the region around *E*_f_ (Fermi level, 0 eV), confirming the typical semiconductor characteristic [48]. After the introduction of X^−^ ions (including F^−^, Cl^−^, and Br^−^), the curves of valence band of β-FeOOH(X)s became more near *E*_f_, leading to the enhanced excitation of charge carriers to the conduction band, showing better electric conductivity. The effect of X^−^ ion embedment on electric conductivity was further analyzed by partial density of states (PDOS) analysis (Fig. S9). It is no doubt that the DOS curves of β-FeOOH are mainly composed with O 2p and Fe 3d states [48] and have the band gap of ~ 2.15 eV. After inducing X^−^ ions into β-FeOOH, because of the hybridization of 2*p* orbit of X^−^, the denser electrons were collected in valence band, resulting in decrease in band gap and improvement in electric conductivity in β-FeOOH(X)s. As displayed in Fig. 4d, the PDOS for Fe 3*d* valance band orbitals (*t*_2g_) of β-FeOOH(X)s shows the larger density than that of β-FeOOH, while the *e*_g_ orbitals of β-FeOOH(X)s show the smaller density after X^−^ embedment, indicating some electrons transferred from *e*_g_ to *t*_2g_ orbitals. This result was also confirmed by solid UV–Vis absorption spectra as shown in Fig. S10. According to the principle of minimum energy, the corresponding electronic configuration for Fe 3d orbitals was changed from $t_{{2{\text{g}}}}^{3} e_{\text{g}}^{2}$ to $t_{{2{\text{g}}}}^{3 + x} e_{\text{g}}^{2 - x - \delta }$, which induced the Jahn–Teller effect in β-FeOOH(X)s, resulting in Fe–O bond change and lattice distortion. The FeO_6_ octahedral tilting changed the charge-transfer energy (∆) from 7.52 eV for β-FeOOH to 6.76 eV for β-FeOOH(F), 7.37 eV for β-FeOOH(Cl), and 7.04 eV for β-FeOOH(Br), respectively, which increased the covalency of Fe-O bond, and influenced the electrochemical activity. (Figs. S11 and S12) These results illustrated that the X^−^ ions had a significant influence on the electronic structure of β-FeOOH and provided a bridge between electronic structure regulation and lattice structural change.

Additionally, not only the electric conductivity but also the wettability of β-FeOOH is influenced by X^−^ ion embedment. To explain this question, the contact angle tests of materials in 1 mol L^−1^ Na_2_SO_3_ electrolyte were carried out. The contact angles decreased from 74.2° for β-FeOOH to 46.0°, 54.2°, and 53.2° for β-FeOOH(F), β-FeOOH(Cl), and β-FeOOH(Br), respectively (Fig. 4a–d). It is indicated that β-FeOOH(X)s had the better wettability in Na_2_SO_3_ electrolyte than that of initial β-FeOOH. This result was consistent with the adsorption energy of obtained materials for SO_3_^2−^ species as provided in Fig. 4e–h. Briefly, the β-FeOOH(F) possessed the lowest adsorption energy, while the β-FeOOH showed the highest, showing the behavior of adsorption for SO_3_^2−^ species is more easily happen on the β-FeOOH(F) electrode, which is the precondition to trigger redox reaction to realize energy conversion and storage. In order to probe why the wettability was enhanced after X^−^ ion embedment, the differential charge density analysis of the fabricated materials was calculated as shown in Fig. 4i. After the introduction of X^−^ ions, the Fe element in β-FeOOH(X) showed the higher valence state than that in initial β-FeOOH. Based on the Mulliken charge analysis (Table S8), the average valence state of Fe element in β-FeOOH(F) was 1.2575, higher than that of β-FeOOH (1.1225), β-FeOOH(Cl) (1.1320), and β-FeOOH(Cl) (1.1775). The existence of high valence state for Fe element was confirmed by XPS measurements. As shown in Fig. 4j–l, the signal of X element in full spectrum confirmed the successful introduction of X^−^ ions into β-FeOOH, and the Fe 2p peak of β-FeOOH(X)s showed the blue shift compared to primal β-FeOOH, indicating the Fe valence state is 3 + *δ* after embedment of X^−^ ions. The high valence state for Fe element facilitates the adsorption of active materials for SO_3_^2−^ species by electrostatic incorporation and endows the electrode materials with good wettability in Na_2_SO_3_ electrolyte. This analysis also provided a bridge between electronic structure regulation and material wettability adjustment. Therefore, because of the improvement for electric conductivity and wettability after X^−^ ion embedment, the designed β-FeOOH(X)s, especially β-FeOOH(F), presented the excellent supercapacitor performances.

### Electrochemical Performances of Dual Electrolyte Asymmetric Supercapacitor

**Fig. 5:**
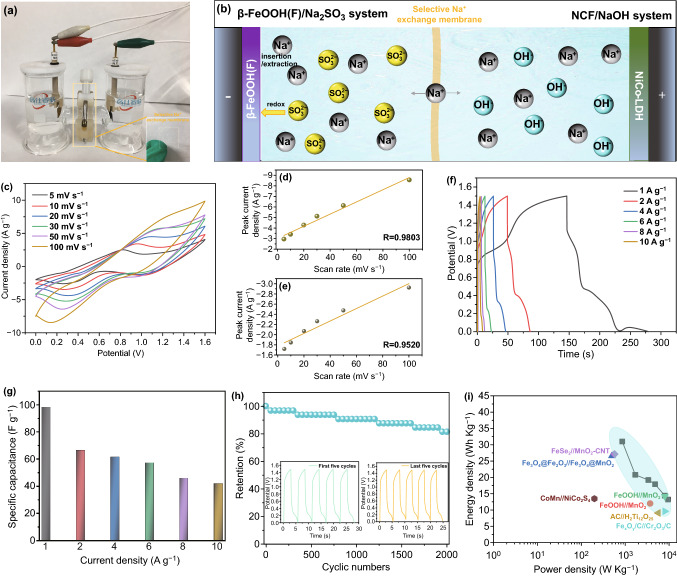
**a** Optical image and **b** scheme of dual electrolyte asymmetric supercapacitor (DESC). **c–i** Electrochemical performances: **c** CVs, **d** peak current density versus scan rate and **e** scan rate^−1/2^, **f** GCDs, **g** rate capacity, **h** cycling stability, inset the first and last five cycles, **i** Ragone plot

To explore the application feasibility of designed β-FeOOH(X) materials, the β-FeOOH(F) was treated as negative electrode in the 1 mol L^−1^ Na_2_SO_3_ solution, while the NiCo hydroxides/Cu(OH)_2_/CF (NCF, the details for the material are shown in SI) as the positive electrode in 1 mol L^−1^ NaOH solution to assemble the unique dual electrolyte asymmetric supercapacitor (DESC) (Fig. 5a, b). Because the two different electrochemical energy storage behaviors happened on positive and negative electrode individually, there are some redox peaks in CV curves attributing to the Ni^3+^/Ni^2+^, Co^3+^/Co^2+^, and SO_3_^2−^/S_2_O_3_^2−^ (Fig. 5c). The linear relationship between peak current density and scan rate confirmed the electrochemical-controlled energy storage process, which is the important characteristic of supercapacitor (Fig. 5d, e). When the current density was 1 A g^−1^, the discharge time was 141.5 s and the specific capacitance was 98.3 F g^−1^ (Fig. 5e). With the current density was increased from 1 to 10 A g^−1^, the specific capacitance of the assembled DESC decreased to 42.1 F g^−1^, showing the good rate capacity (42.8%, Fig. 5f, g). The cycling stability was investigated by repeated charge/discharge processes at 10 A g^−1^ current density (Fig. 5h). After 2000 charge/discharge cycles, the DESC device retained 81.54% of its initial capacitance value, displaying excellent cycling stability. The specific energy density of the DESC device was about 31.0 Wh kg^−1^ at a power density of 858.5 W kg^−1^, and remained as high as 13.2 Wh kg^−1^ even at a high power density of 9504.0 W kg^−1^, which are comparable to the values reported in the previous literature (Fig. 5i) [3, 49–54], verifying the application feasibility of the assembled DESC device.

## Conclusion

In summary, we demonstrated a series of halogen ion doping β-FeOOH with enhanced supercapacitor negative electrode performances. DFT calculations and structure characterizations confirmed that the incorporation of X^−^ ion led to the change of Fe–O bond length and structural distortion of β-FeOOH, resulting in the relatively narrow band gap. Because of the strong electronegativity of X^−^, the Fe element in β-FeOOH(X)s presented the unexpected high valence state (3 + *δ*), which is enhancing the capability for adsorbing SO_3_^2−^ species. Benefiting from these unique characteristics, the β-FeOOH(X)s showed the excellent supercapacitor negative electrode performances. The present work not only affords an efficient strategy for controlling and activating the intrinsic supercapacitor properties of negative electrode material, but also provides an in-depth insight into the mechanism of the enhanced electrochemical performances from tuning electronic structure.

## Electronic Supplementary Material

Below is the link to the electronic supplementary material.

Supplementary material 1 (PDF 1321 kb)
