# Supplementary material for: The Principle of Introducing Halogen Ions Into β-FeOOH: Controlling Electronic Structure and Electrochemical Performance
Source: Nanomicro Lett. 2020 May 6;12:107. doi: 10.1007/s40820-020-00440-2 (PMC7770722; doi:10.1007/s40820-020-00440-2)
Supplement: Supplementary file 1 — Supplementary material 1 (PDF 1321 kb) [file 40820_2020_440_MOESM1_ESM.pdf]

Supporting Information for

The principle of Introducing Halogen Ions Into β-FeOOH: Controlling Electronic Structure and Electrochemical Performance

Dongbin Zhang[1], Xuzhao Han[1], Xianggui Kong[1, *], Fazhi Zhang[1], Xiaodong Lei[1, *]

[1]State Key Laboratory of Chemical Resource Engineering, Beijing University of Chemical Technology, PO Box 98, Beijing 100029, People's Republic of China

*Corresponding authors. E-mail: leixd@mail.buct.edu.cn (X. Lei); kongxg@mail.buct.edu.cn (X. Kong)

Supplementary Figures and Tables

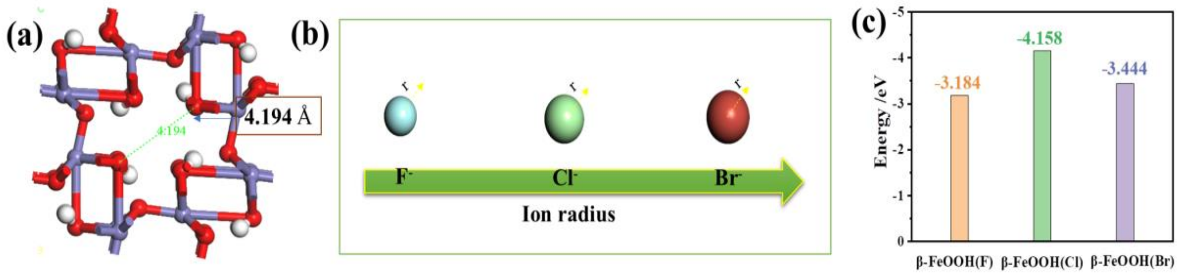

Fig. S1 a Crystal structure of β-FeOOH; **b** radius of halogen anions for F^-, Cl^- and Br^-; **c** the adsorption energy of β-FeOOH for halogen anions, including F^-, Cl^-, and Br^-

Table S1 Comparsion of different halide ions radius

Ion	Bare ion radius (Å)	Hydrated radius (Å)
F^-	1.16	3.52
Cl^-	1.64	3.32
Br^-	1.80	3.30

Table S2 Comparision of standard electrode potentials

Redox reaction	Potential
$Fe^{3+} + e \longrightarrow Fe^{2+}$	+0.77
$F_2 + 2e \longrightarrow 2F^-$	+2.87
$Cl_2 + 2e \longrightarrow 2Cl^-$	+1.36
$Br_2 + 2e \longrightarrow 2Br^-$	+1.07
$I_2 + 2e \longrightarrow 2I^-$	+0.54

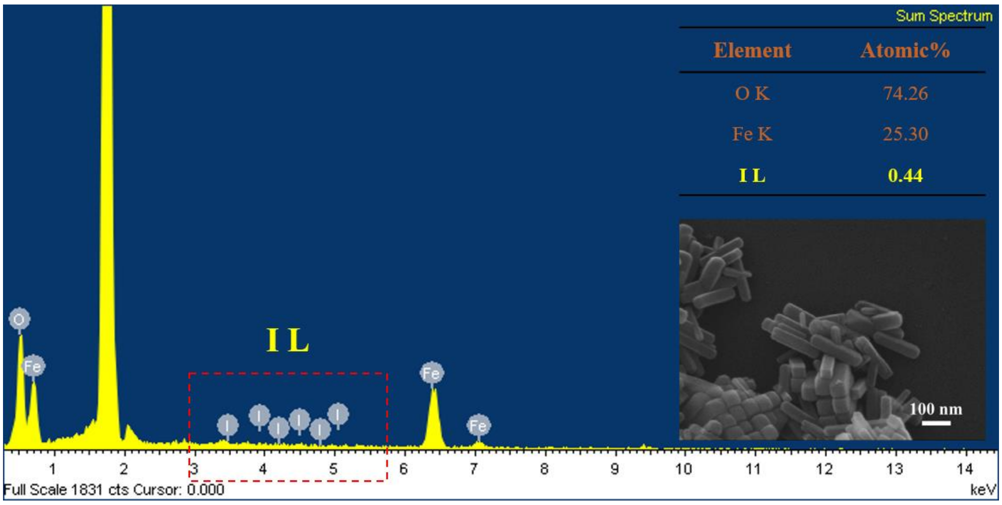

Fig. S2 The EDS of β-FeOOH(I), inset the corresponding SEM image

Table S3 Comparison of the length of different Fe-Os bond

Sample	Fe-O1	Fe-O2	Fe-O3	Fe-O4	Fe-O5	Fe-O6	Fe-O7	Fe-O8
β-FeOOH	1.957	2.024	3.003	2.407	1.957	2.015	3.115	2.450
β-FeOOH(F)	1.952	2.117	2.504	2.428	1.883	1.995	2.488	2.543
β-FeOOH(Cl)	1.967	1.923	2.168	2.664	2.285	2.207	2.809	3.041
β-FeOOH(Br)	1.844	2.109	2.099	1.953	2.682	1.870	2.873	2.989

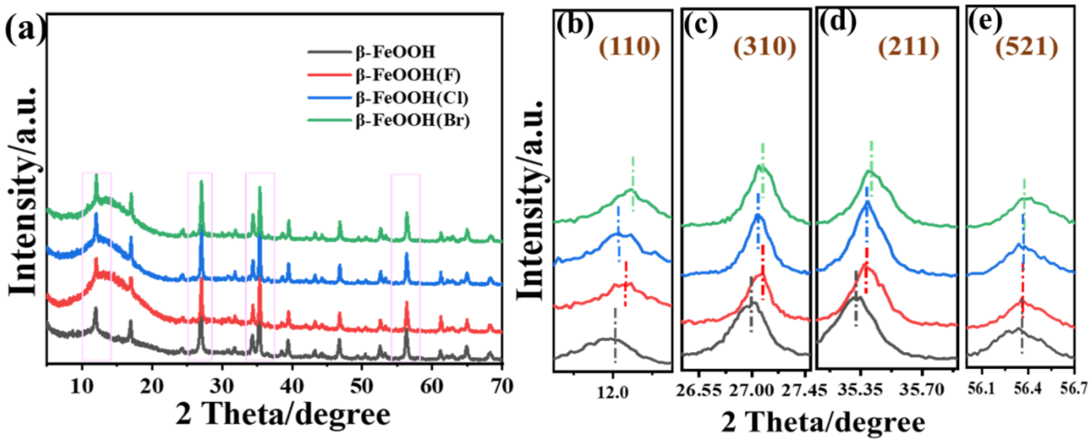

Fig. S3 XRD results of β-FeOOH and β-FeOOH(X)s

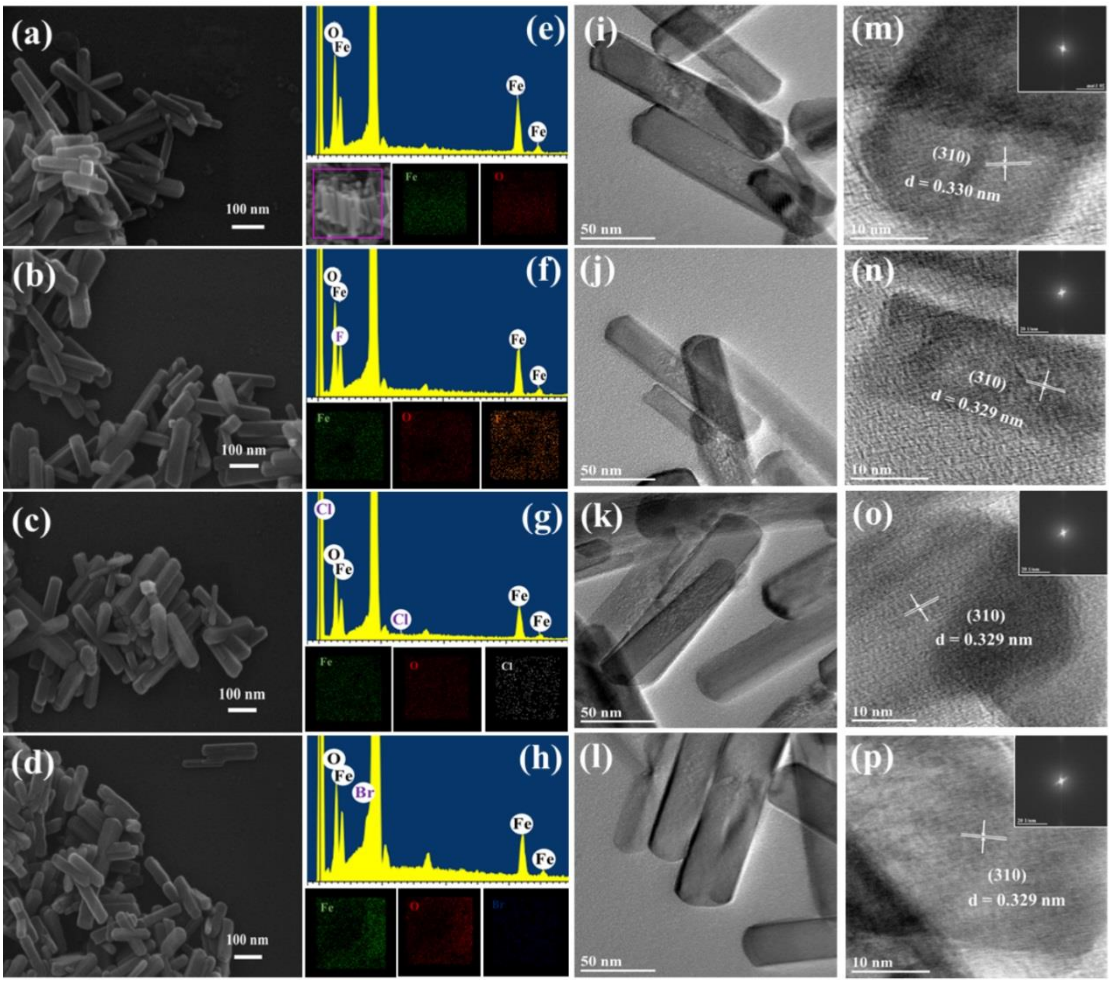

Fig. S4 SEM and HRTEM images of β-FeOOH, β-FeOOH(F), β-FeOOH(Cl) and β-FeOOH(Br). **a-d** SEM images; **e-h** EDS and mapping images; **i-p** HRTEM images, inset the FFT images

Table S4 EDS results of samples

**Samples**	**Elements**	**Atomic%**
**β-FeOOH**	**Fe**	25.73
	**O**	74.27
	**---**	---
**β-FeOOH(F)**	**Fe**	22.17
	**O**	64.13
	**F**	13.70
**β-FeOOH(Cl)**	**Fe**	13.79
	**O**	75.54
	**Cl**	10.67
**β-FeOOH(Br)**	**Fe**	13.96
	**O**	75.35
	**Br**	10.69

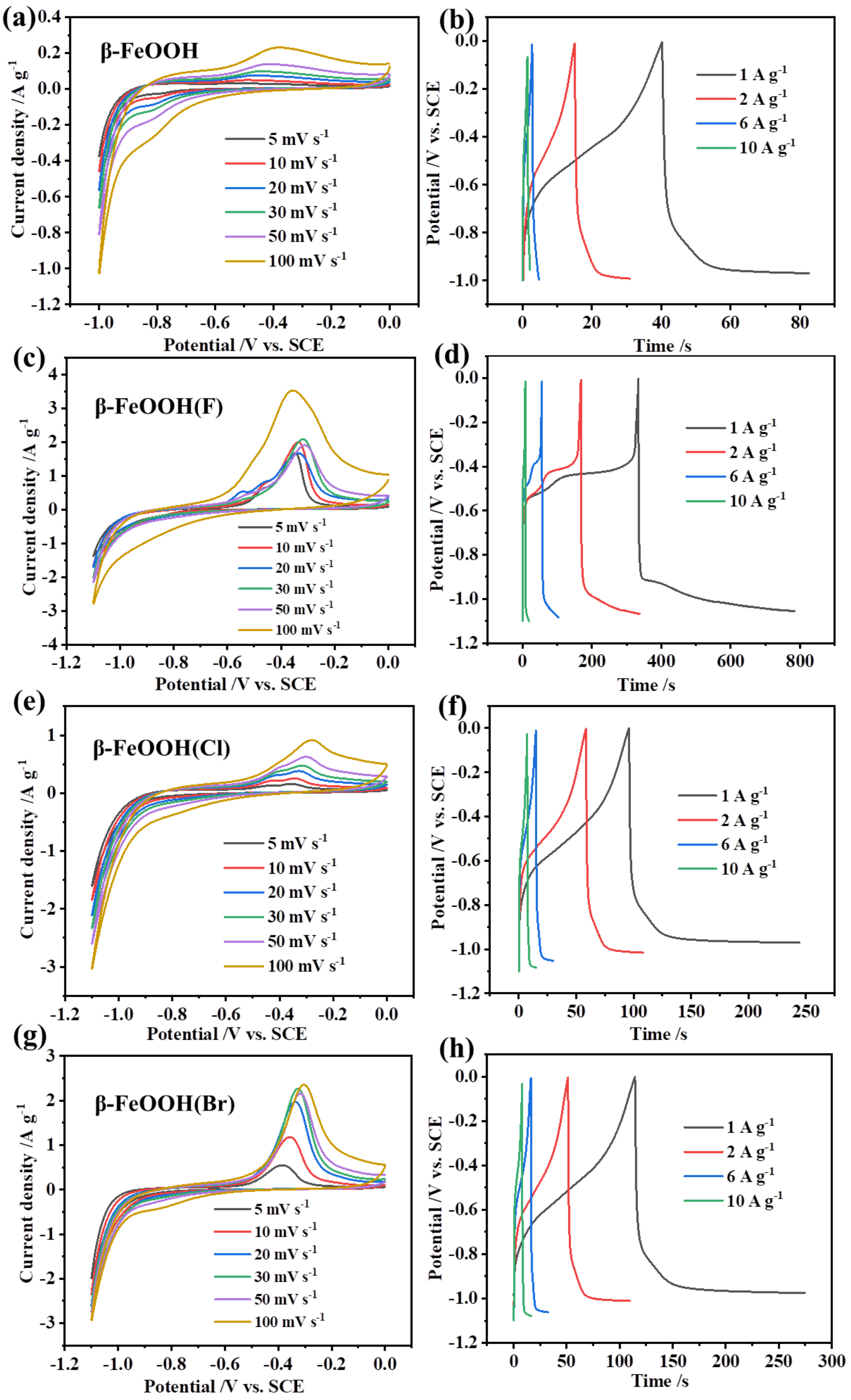

Fig. S5 The CV and GCD of **a, b** β-FeOOH; **c, d** β-FeOOH(F); **e, f** β-FeOOH(Cl) and **g, h** β-FeOOH(Br)

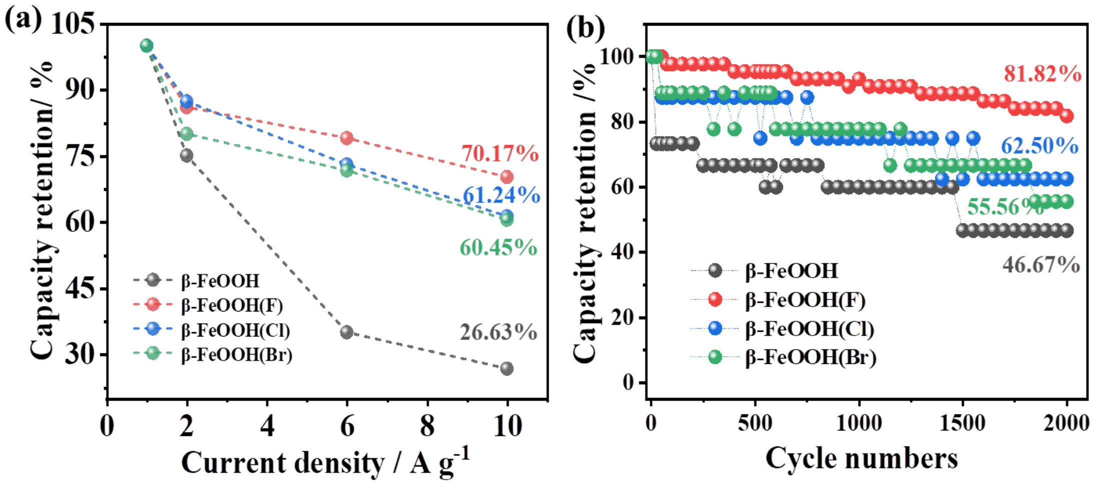

Fig. S6 a Rate capacity and **b** cyclic stability of β-FeOOH, β-FeOOH(F), β-FeOOH(Cl) and β-FeOOH(Br)

Table S5 Comparison of electrochemical performances of FeOOH-based electrodes

Materials	Potential Window/V	Specific Capacitance	Rate Capacity/%	Cyclic Stability/%	Refs.
β-FeOOH(F)	-1.1-0 V	391.9 $F\cdot g^{-1}$ at 1 A g^{-1}	70.17% from 1 to 10 A g^{-1}	80.82% after 2000 cycles	This work
FeOOH nanorod	-1.08- 0 V	396 $F\cdot g^{-1}$ at 0.5 A g^{-1}	64% from 0.5 to 10 A g^{-1}	83% after 500 cycles	[S1]
β-FeOOH	-0.85- -0.1 V	116 $F\cdot g^{-1}$ at 0.5 A g^{-1}	80% from 0.5 to 1.5 A g^{-1}	Not give	[S2]
Metal-FeOOH	-1- -0.6 V	463.18 $F\cdot g^{-1}$ at 0.1 A g^{-1}	~20% from 0.11 to 10 A g^{-1}	96.36% after 1000 cycles	[S3]
FeOOH/RGO	-0.8- 0 V	142.0 $F\cdot g^{-1}$ at 1 A g^{-1}	90% from 1 to 40 A g^{-1}	~90% after 1000 cycles	[S4]
Fe_3O_4/FeOOH	-1.1- 0 V	300 $F\cdot g^{-1}$ at 2 mV s^{-1}	~25% from 2 to 250 mV s^{-1}	~80% after 150 cycles	[S5]
Amorphous FeOOH/$Ti_3C_2T_x$	-0.8- 0 V	217 $F\cdot g^{-1}$ at 1 A g^{-1}	64% from 1 to 12 A g^{-1}	82% after 3000 cycles	[S6]

FeOOH@ SnO_2	-0.7- -0.2 V	7.013 mF·cm^{-2} at 0.20 mA cm^{-2}	32.22% from 0.20 to 2.26 mA cm^{-2}	82.8% after 2000 cycles	[S7]

Table S6 Concentration of Fe element in electrolyte before and after electrochemical tests, when the β-FeOOH(F) as the working electrode

**Fe elements**	**Conc. (μg L^{-1})**
**Before electrochemical tests**	**2.630**
**After electrochemical tests**	**2.528**

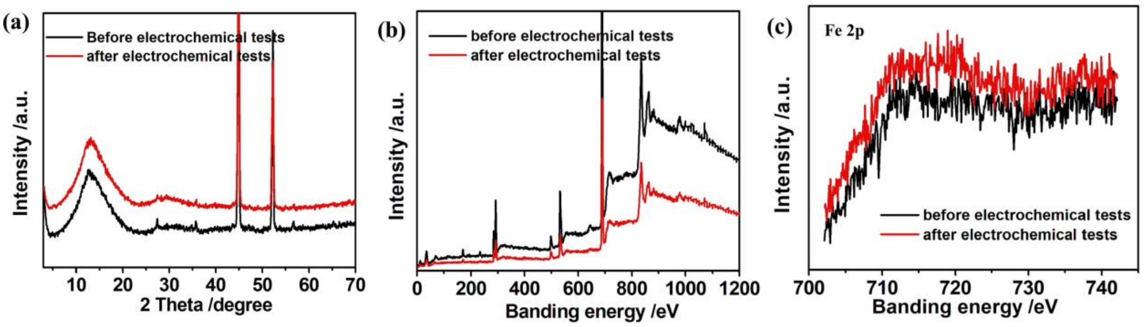

Fig. S7 The XRD **a** and XPS **b, c** measurements of β-FeOOH(F) electrode before and after electrochemical tests

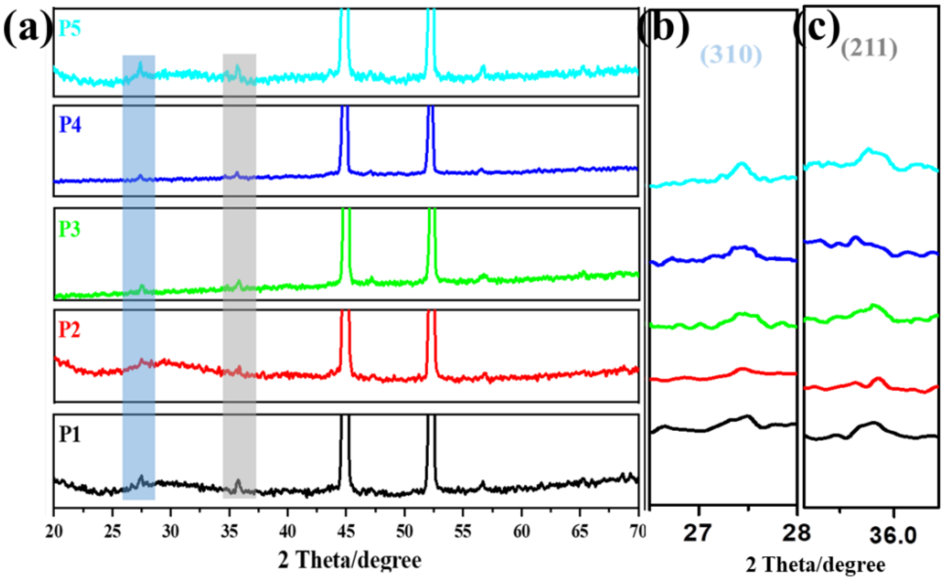

Fig. S8 ex-XRD tests of β-FeOOH(F) electrode during the charge and discharge test

Table S7 XPS test results of β-FeOOH(F) under different charge and discharge potentials

Name		Position	%At Conc
Na 1s	P1	1072.60	1.46
	P2	1072.63	1.44
	P3	1072.32	**0.70**
	P4	1072.38	2.44
	P5	1072.74	2.57
**Name**		**Position**	**%At Conc**
Fe 2p	P1	712.28	4.10
	P2	712.40	4.29
	P3	**712.73**	4.41
	P4	712.54	4.22
	P5	712.02	4.13

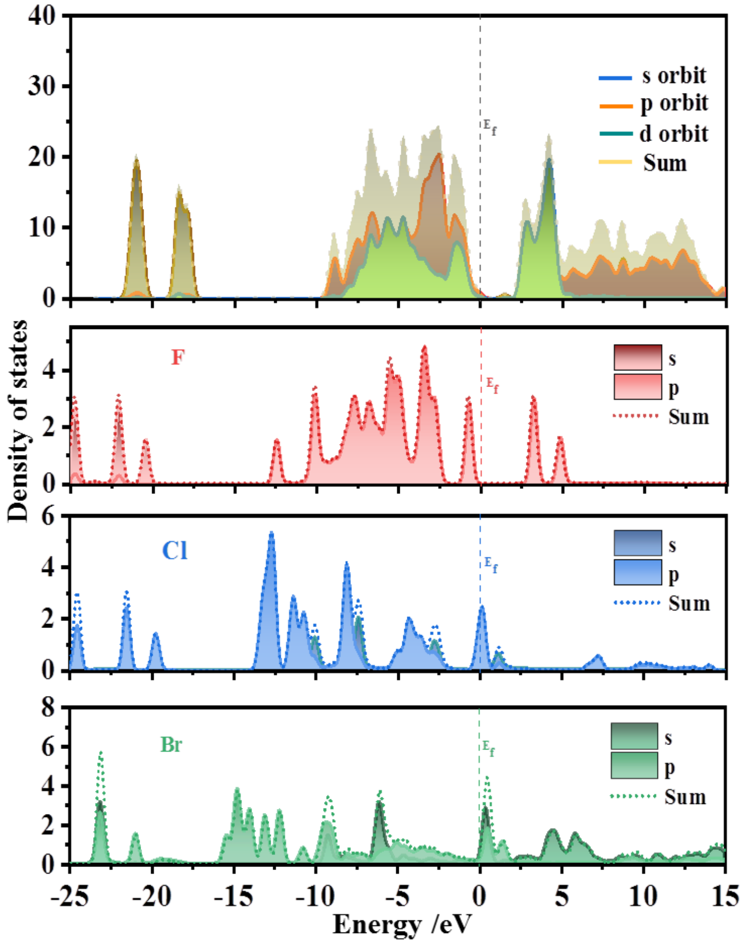

Fig. S9 PDOS of β-FeOOH and β-FeOOH(X)s

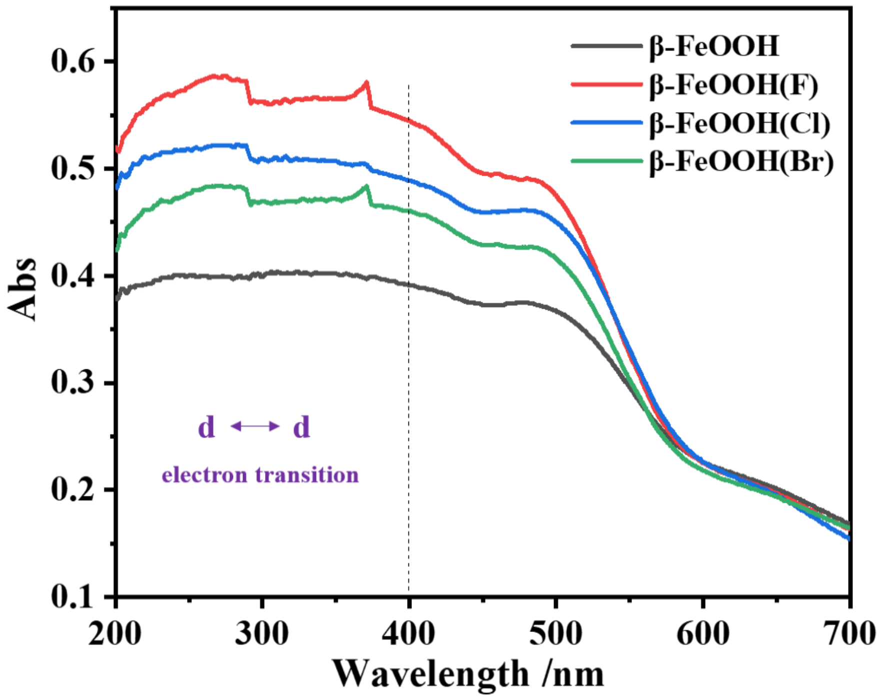

Fig. S10 Solid UV-vis absorption spectra of β-FeOOH and β-FeOOH(X)s

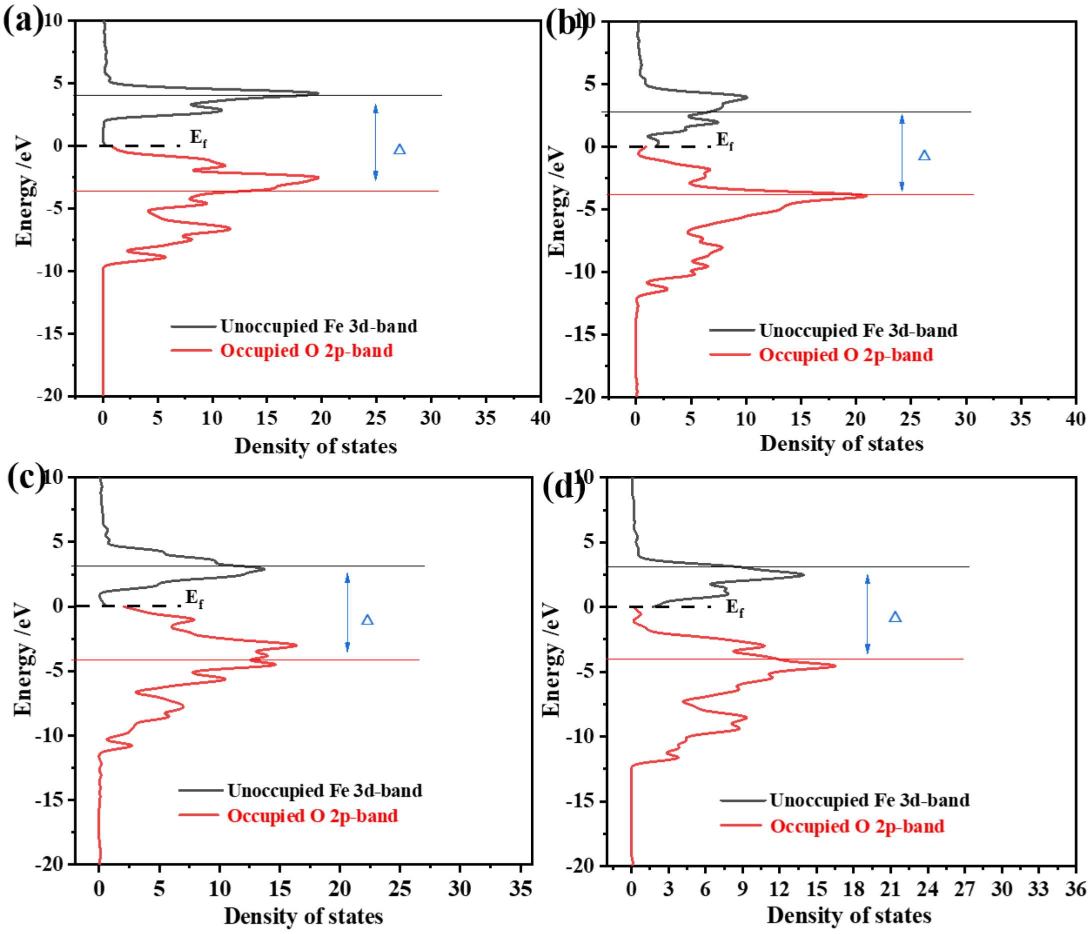

Fig. S11 Illustration of charge-transfer energy of **a** β-FeOOH, **b** β-FeOOH(F), **c** β-FeOOH(Cl) and **d** β-FeOOH(Br)

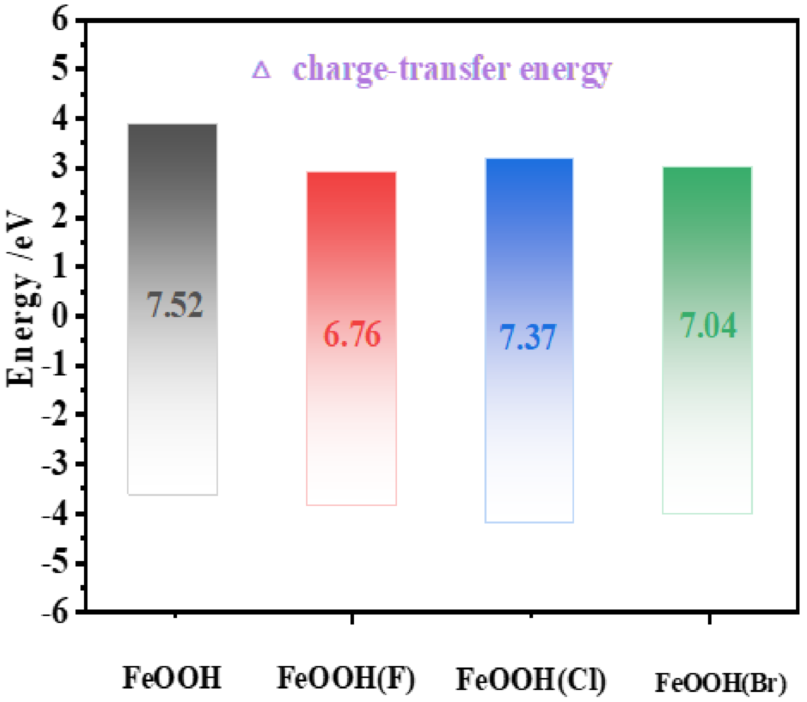

Fig. S12 Comparison of charge-transfer energy of β-FeOOH and β-FeOOH(X)s

Table S8 Mulliken charge analysis

**Sample**	**β-FeOOH**	**β-FeOOH(F)**	**β-FeOOH(Cl)**	**β-FeOOH(Br)**
**Fe1**	1.26	1.14	1.10	1.15
**Fe2**	1.09	1.22	1.12	1.19
**Fe3**	1.03	1.43	1.23	1.17
**Fe4**	1.11	1.24	1.08	1.20
**Average**	**1.1225**	**1.2575**	**1.1320**	**1.1775**

Supplementary References

[S1]J. Li, D. Chen, Q. Wu, X. Wang, Y. Zhang, Q. Zhang, FeOOH nanorod arrays aligned on eggplant derived super long carbon tube networks as negative electrodes for supercapacitors. New J. Chem. **42**(6), 4513-4519 (2018). https://doi.org/10.1039/c7nj04662f

[S2]W.-H. Jin, G.-T. Cao, J.-Y. Sun, Hybrid supercapacitor based on MnO_2 and columned FeOOH using Li_2SO_4 electrolyte solution. J. Power Sources **175**(1), 686-691 (2008). https://doi.org/10.1016/j.jpowsour.2007.08.115

[S3]R. Barik, B. K. Jena, M. Mohapatra, Metal doped mesoporous FeOOH nanorods for high performance supercapacitors. RSC Adv. **7**(77), 49083-49090 (2017). https://doi.org/10.1039/c7ra06731c

[S4]H.-W. Chang, C.-L. Dong, Y.-R. Lu, Y.-C. Huang, J.-L. Chen et al., X-ray

absorption spectroscopic study on interfacial electronic properties of FeOOH/reduced graphene oxide for asymmetric supercapacitors. ACS Sustain. Chem. Eng. **5**(4), 3186-3194 (2017). https://doi.org/10.1021/acssuschemeng.6b02970

[S5]L. O'Neill, C. Johnston, P. S. Grant, Enhancing the supercapacitor behaviour of novel Fe_3O_4/FeOOH nanowire hybrid electrodes in aqueous electrolytes. J. Power Sources **274**, 907-915 (2015). https://doi.org/10.1016/j.jpowsour.2014.09.151

[S6]X. Zhang, Y. Liu, S. Dong, Z. Ye, Y. Wei, Low-temperature synthesized nanocomposites with amorphous feooh on $Ti_3C_2T_x$ for supercapacitors. J. Alloy. Compd. **744**, 507-515 (2018). https://doi.org/10.1016/j.jallcom.2018.02.080

[S7]R. Li, X. Ren, F. Zhang, C. Du, J. Liu, Synthesis of Fe_3O_4@SnO_2 core-shell nanorod film and its application as a thin-film supercapacitor electrode. Chem. Commun. **48**(41), 5010-5012 (2012). https://doi.org/10.1039/c2cc31786a
